# Dizziness Handicap Inventory in Clinical Evaluation of Dizzy Patients

**DOI:** 10.3390/ijerph18052210

**Published:** 2021-02-24

**Authors:** Ewa Zamyslowska-Szmytke, Piotr Politanski, Magdalena Jozefowicz-Korczynska

**Affiliations:** 1 Balance Disorders Unit, Nofer Institute of Occupational Medicine, 91-348 Lodz, Poland; 2 Nofer Institute of Occupational Medicine, 91-348 Lodz, Poland; piotr.politanski@imp.lodz.pl; 3 Balance Disorders Unit, Department of Otolaryngology, Medical University of Lodz, 90-237 Lodz, Poland; magdakoras@wp.pl

**Keywords:** DHI, vertigo, psychogenic dizziness, migraine, positional vertigo, vestibular dysfunction

## Abstract

(1) Objectives: The evaluation of dizzy patients is difficult due to nonspecific symptoms that require a multi-specialist approach. The Dizziness Handicap Inventory (DHI) is widely used in the assessment of dizziness-related disability, but its clinical efficacy needs further expansion. The aim of this study was to identify the subscales of DHI that may correlate with some vestibular or nonvestibular dysfunctions. (2) Material and methods: This observational study included 343 dizzy patients with one of the following clinical conditions: Vestibular impairment noncompensated or compensated, central or bilateral, benign paroxysmal positional vertigo (BPPV), migraine and psychogenic dizziness. Principal component analysis was used to examine the factorial structure of the questionnaire. (3) Results: The DHI questionnaire total scoring and its vestibular subscale distinguished between patients with compensated and uncompensated vestibular dysfunction with positive predictive values of 76% and 79%, respectively. The DHI items composing the F3 (positional) subscale revealed the highest scoring in the BPPV group with 75% sensitivity and 92% negative predictive value (NPV) in reference to Dix–Hallpike tests. The DHI total score and the subscales scores correlated with anxiety-depression, and the highest correlation coefficients were calculated for vestibular (F2 0.56) and anxiety (F5 0.51) subscales. (4) Conclusions: Our analysis revealed that the DHI vestibular subscale distinguishes between patients with compensated and uncompensated vestibular dysfunction. The positional subscale showed the highest scoring in the BPPV group with high sensitivity and low specificity of the test. The DHI is highly correlated with patients’ psychological status.

## 1. Introduction

Dizziness and/or vertigo are the most common reported medical complaints affecting 15–35% of the adult population dependent on the study group [1,2]. Subjects reporting dizziness describe a range of sensations, such as feeling faint, woozy, weak, or unsteady. According to the Classification of Vestibular Disorders of the Barany Society, dizziness is defined as a nonvertiginous sensation of disturbed or impaired spatial orientation without a false or distorted sense of motion [3]. Diagnosis is usually difficult because these complaints are nonspecific and the differential diagnosis is broad. Consequently, dizziness is a cause of disability and inability to work. Primary care is the first point of contact for dizzy patients [4]. A self-reported questionnaire could be of great help in evaluating the clinical status of the patient. There are several questionnaires for vertigo and dizziness handicap assessment, e.g., Vestibular Disorders of Daily Living Scale (VADL); Activities-specific Balance Confidence (ABC); Vertigo Handicap Questionnaire (VHQ); Vertigo, Dizziness, Imbalance Questionnaire (VDI); UCLA-DQ; UCLA Dizziness Questionnaire.

The Dizziness Handicap Inventory (DHI) is one of the most popular questionnaires for assessment of the dizziness handicap [5]. The DHI was developed by Jacobson and Newman to assess disability grade [6]. The DHI consists of 25 items designed to determine dizziness-dependent changes grouped into three domains: Functional, emotional, and physical. Some studies that assessed patients with vestibular impairment used domains that differed from the original ones [7,8]. The DHI was originally developed in the English language for USA patients, but in the past decades, it has been translated and validated in many other languages, e.g., to German [9] or Spanish [10]. However, it has been observed that this questionnaire yields limited conclusions related to the clinically important information [8,10,11]. In the literature, a few studies have already concentrated on the relationship between the DHI score and (1) selected diseases or clinical status [12,13], and (2) vestibular testing objective methods [14,15].

Clinical status. The DHI is used mainly as a measure of handicap in different diseases, but few studies consider its usefulness as a disease indicator. The association between DHI and clinical status of the patients was analyzed by Hansson et al. [13] in subgroups of patients with multisensory dizziness, chronic peripheral vestibular disorder, whiplash-associated disorder, unspecific dizziness, phobic postural vertigo, and dizziness of cervical origin. The group with phobic postural vertigo had the highest total score of DHI, while the vestibular group had the lowest one. Graham et al. [16] investigated the relationship between total DHI scores and the presence of the structural, functional, and psychiatric disorders. They found that the categories of illnesses had large effects on total DHI scores. Structural disorders have caused lower scores than functional and psychiatric ones. Whitney et al. [12] proposed using a subscale composed of five DHI items for the diagnosis of benign paroxysmal positional vertigo (BPPV) with a sensitivity of 81% and low specificity of 34%. Two of the five items strongly correlated with the BPPV (“getting out of bed” and “rolling over in bed”). Similar items have been included in the motion-provoked dizziness subscale in the Vestibular Rehabilitation Benefit Questionnaire [17].

Patients showing signs of dizziness often have comorbid mental symptoms. For instance, panic disorder is highly prevalent in patients presenting with vestibular symptoms [18]. Moreover, anxiety and depression are diagnosed with greater frequency in dizzy patients than in the nondizzy population [19].

Testing methods. To date, the association between the objective vestibular tests and DHI scores is poorly understood. Vestibular symptoms and the DHI scores have been demonstrated to be significantly negatively correlated with the scores of the Sensory Organization Test (SOT) of the dynamic posturography [6,20]. Gill-Body [14] showed a correlation between DHI and SOT, which concerned only the third SOT condition (sway-referenced visual surround motion during stable platform condition) and the emotional subscale of DHI. Yip and Strupp [15] could not find a significant correlation between the DHI score and caloric test parameters, video head impulse-test results, or vestibular-evoked potentials measure of otolith function.

The aim of our present study was to assess the DHI results obtained from a cohort of patients with vestibular and nonvestibular signs using factor analysis. We reasoned that this type of analysis could potentially identify individual subscales of the DHI, which would correlate with patients’ clinical status, e.g., compensation level, positional vertigo, anxiety, and depressive symptoms. In addition, we carried out comparative analysis of the results between the groups of patients with vestibular and nonvestibular vertigo or dizziness complaints.

## 2. Materials and Methods

This observational study was approved by the Ethics Committee (No. 17/2014). All patients signed the informed consent.

We recruited consecutive patients referred primarily for the diagnosis of vertigo/dizziness. No subjects with acute vestibular loss were recruited. The sample included 628 subjects who underwent the following diagnostic procedures: Collecting detailed clinical history, complete neuro-otological bedside examination, and laboratory tests battery: Tympanometry, pure-tone audiometry, video head impulse test (Interacoustics), and videonystagmography (VNG) tests recorded with an Ulmer device (SYNAPSIS). The following VNG tests were performed: Spontaneous and gaze-evoked nystagmus, oculomotor tests (OMTs)-saccades, pursuit, optokinetic, caloric test by Fitzgerald–Hallpike, and rotational chair tests (sinusoidal pendular rotation at frequencies 0.04, 0.08, 0.1, 0.32, and 0.64 Hz). Neurological consultation and MRI imaging were obtained in patients with neurological signs or for the differential diagnoses.

From the initial 628 patients, 285 with signs of two or more confirmed or suspected diseases were excluded (multi-diseases patients), e.g., migraine and positional vertigo, dysfunction of the central and peripheral part of vestibular system.

For factor analysis, 343 patients were included. The main criterion was persisting vertigo and/or dizziness and only one type of vestibular system dysfunction (peripheral or central), established based on clinical examination and VNG tests results. Peripheral vestibular impairment or loss was diagnosed when the canal paresis was >19% and no abnormalities were found in OMTs. The central vestibular dysfunction was diagnosed when the morphology and caloric responses were in a normal range and OMTs revealed abnormal recordings.

The mean age of our cohort was 54.3 ± 14.6 (mean ± SD); range 20–87 years, including 248 women and 95 men.

**Figure 1:**
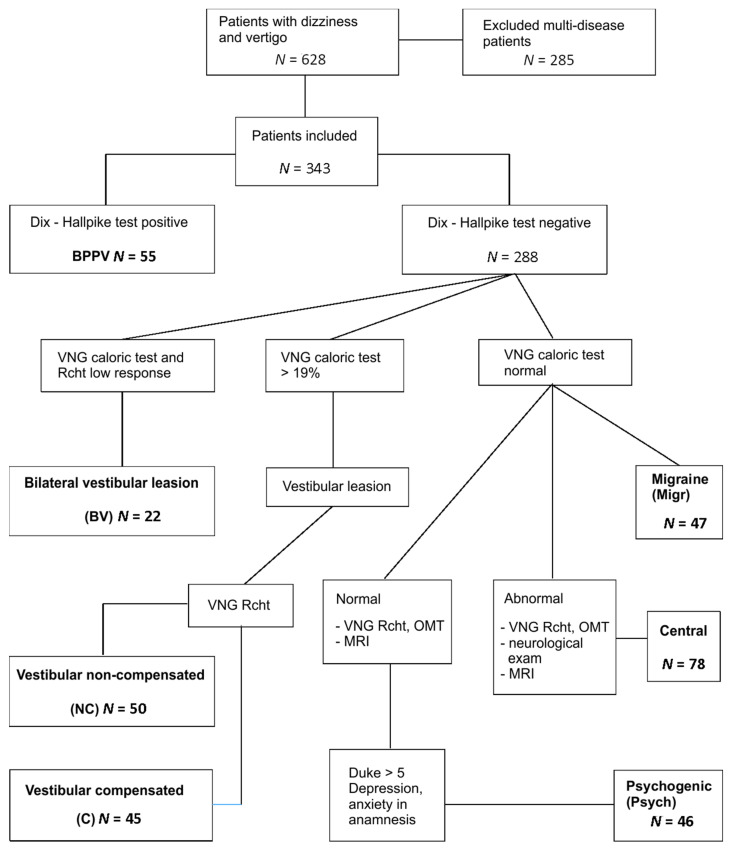
Flowchart of diagnostic procedures and outcomes. BPPV—benign paroxysmal positional vertigo; VNG—videonystagmography; OMT—oculomotor tests; Rcht—rotational chair tests, MRI—magnetic resonance imaging.

The study groups in the cohort were identified based on the following (Figure 1):

- 55 BPPV (benign paroxysmal positional vertigo) subjects: Positive Dix–Hallpike test, VNG—no vestibular and central signs.
- 50 vestibular noncompensated (NC): VNG canal paresis >19% in caloric test, directional preponderance (DP) > 2°/s in rotational tests (for more than one frequency of rotation), phase lead > 20° for low frequencies of rotation.
- 45 vestibular compensated (C): VNG canal paresis >19%, no DP in rotational tests, phase lead only slightly increased or not at all.
- 22 bilateral vestibular (BV): VNG caloric reactivity < 10°/s, rotational test’s VOR gains absent in low frequencies of rotation (0.04 Hz, 0.08 Hz)
- 47 migraine (Migr): VNG canal paresis ≤ 19%, headaches that fulfil the criteria of vestibular migraine or migraine with brainstem aura, according to International Headache Classification, version 3.
- 78 central (Central): VNG caloric canal paresis ≤ 19%, abnormal oculomotor tests (OMTs) results: Latency and precision in saccades, morphology and gain in smooth pursuit, and optokinetic tests and/or high caloric reactivity and abnormal fixation in caloric/rotational tests and/or directional preponderance in rotational chair tests. Patients with neurological diseases but with normal VNG were not included.
- 46 psychogenic (Psych): VNG—no vestibular or central pathology, increased scoring of Duke anxiety and depression scale (>5), depression and/or anxiety episodes treatment currently or in the past; features of phobic postural vertigo or chronic subjective dizziness; 3 patients fulfilled the criteria of persistent postural-perceptual dizziness (PPPD) [21].

The BPPV, C, and NC groups were homogenous. The BPPV group included patients with active BPPV confirmed by the Dix–Hallpike test. In the patients with peripheral vestibular dysfunction, the noncompensated and compensated groups had a medical history of vestibular neuritis. The compensation was confirmed by the VNG rotational tests.

The patients included in the Central group were inhomogeneous. The Central group included dizzy patients referred to the clinic after neurological examination and brain imaging. They were diagnosed with transient ischemic attacks, stroke, multiple sclerosis, and others.

The group of the psychogenic dizziness enclosed patients with no VNG abnormalities, with normal neuro-otological examination and no other vestibular/positional episodes in the last half-year. The most common symptoms in the psychogenic dizziness group were anxiety, fear of falling, hypersensitivity to motion stimuli, long-lasting dizziness, and instability.

There were no age differences between the groups, except for those with migraines, who were significantly younger than the remaining ones.

The DHI was self-completed by each patient before vestibular testing and medical interview. In addition, patients completed the Duke Anxiety and Depression questionnaire. This scale consists of three items concerning anxiety and four items for depression.

The total score ranges from 0 to 14 and an abnormal value is over 5 points (Polish validated version can be found at https://eprovide.mapi-trust.org/instruments/duke-anxiety-depression-scale02.2015 (accessed on 24 February 2021)).

### Data Analysis

To evaluate different dimensions of the DHI, principal component analysis (PCA) was performed. PCA is concerned with establishing which linear components exist within the data and how a particular variable might contribute to that component. The PCA was conducted with all 25 items with oblique rotation. Rotation was included into analysis to maximize the loadings of the variables onto one factor (the factor that intersects the cluster) and minimize them on the remaining factor(s). The oblique rotation was chosen over orthogonal rotation as there were good reasons to suppose that the underlying factors could be related in theoretical terms. The pre-test assumptions were fulfilled (Bartlett’s test was highly significant and the Kaiser–Meyer–Olkin (KMO) measure of sampling adequacy >0.75). Bartlett’s test tells us whether the analyzed correlation matrix is significantly different from an identity matrix. Therefore, if it is significant, it means that the correlations between variables are significantly different from zero. The KMO can be calculated for individual and multiple variables and represents the ratio of the squared correlation between variables to the squared partial correlation between variables. The KMO statistic varies between 0 and 1. A value of 0 indicates that the sum of partial correlations is large relative to the sum of correlations, indicating diffusion in the pattern of correlations (hence, factor analysis is likely to be inappropriate). A value close to 1 indicates that patterns of correlations are relatively compact and so factor analysis should yield distinct and reliable factors [22].

An initial analysis has been run to obtain eigenvalues for each component in the data. In the analysis, factors >1 (Kaiser’s K-1 criterion) were extracted. This criterion is based on the idea that the eigenvalues represent the amount of variation explained by a factor and that an eigenvalue of 1 represents a substantial amount of variation. The factors structure was identified using the oblique Promax rotation with Kaiser normalization. Item loadings were evaluated in line with the proposals from Kurre et al. [7] (>0.6 on four or more variables) and Tamber et al. [8] (loadings ≥ 0.32). Thus, item loadings ≥0.4 on a minimum of 3 variables were included.

Exploratory factor analysis was used to check dimensionality; then, Cronbach’s alpha was used as a measure of the internal consistency of subscales. It is considered to be a measure of the reliability. A Cronbach’s alpha coefficient of 0.70 or higher is considered acceptable.

The analysis of the association between the DHI results and the Duke Anxiety and Depression Scale was performed using Pearson’s correlation coefficients. One-way ANOVA and Bonferroni post-hoc tests were used to assess the differences in the mean age or the mean DHI total score of the clinical groups. 

## 3. Results

### 3.1. Factor Analysis

Bartlett’s test of sphericity was highly significant (*p* < 0.0000), indicating that correlations between the items were sufficiently high for PCA analysis. The Kaiser–Meyer–Olkin measure verified the sampling adequacy for the analysis, and KMO resulted in a value of 0.924, which is in agreement with the recommended assumptions (‘superb’ according to Field [22]).

An initial analysis was run to obtain eigenvalues for each component in the data. Five components had eigenvalues over Kaiser’s criterion of 1 and this combined explains 58% of the variance. With accordance to the scree plot, this is the number of components that have been retained in the final analysis. Table 1 shows the factor loadings after rotation. As every factor consisted of a minimum of three variables whose loading was above 0.4 (the minimal criterion), this solution has been investigated. Cronbach’s alpha for every single factor was acceptable.

The items clustering on the same components suggest that Factor 1 (consisting of six items) assesses restriction in participation (travel, walking, staying home alone) with the weak input of family relationships. Factor 2 represents activities aggravating vestibular symptoms (5 items). Factor 3 contains five items characteristic for positional vertigo. Factors 4 and 5 are connected to handicap and depression/anxiety, respectively. There were two items that overlapped F2 and F3 and were excluded from further analysis.

The correlation coefficient between total DHI and the Duke anxiety and depression questionnaire was statistically significant but fair (0.37). The Duke questionnaire was correlated to F1–F5 and the highest correlation coefficients were calculated for F2 (0.56) and F5 (0.51), lower being for F3 (0.43), F1 (0.40), and F4 (0.35).

### 3.2. Analysis in Clinical Subgroups

**Figure 2:**
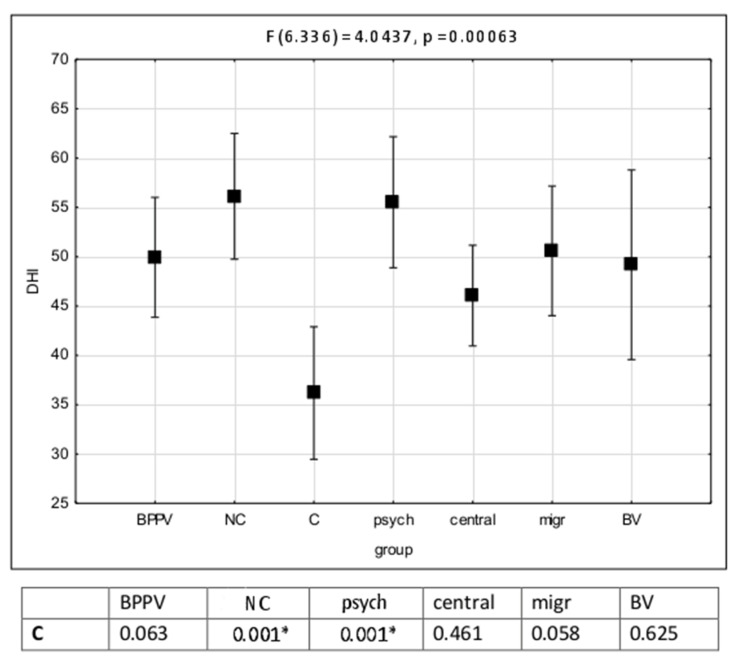
Dizziness Handicap Inventory (DHI) total scores distribution in clinical groups. BPPV—benign paroxysmal positional vertigo. NC—vestibular noncompensated group. C—vestibular compensated group. Migr—migraine group. BV—bilateral vestibular impairment. Psych—psychogenic dizziness. Post-hoc Bonferroni analysis results in the bottom of the figure. The statistically significant differences between groups were mark with asterisk (*p* < 0.05).

The analysis of mean values of the DHI total scores revealed the lowest values in the compensated (C) subgroup. Statistically significant differences were found between mean scores of the C subgroup and the NC and psychogenic dizziness groups (Figure 2).

The analysis of the intergroup relations in each of the five factors revealed several relationships. There were no intergroup differences in item sets comprising factor 1. In Factor 2, the compensated group revealed markedly lower scoring and statistically significant differences as compared to the noncompensated, psychogenic, and central groups.

**Figure 3:**
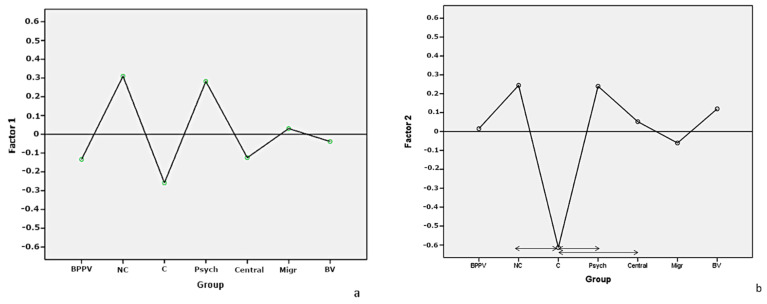

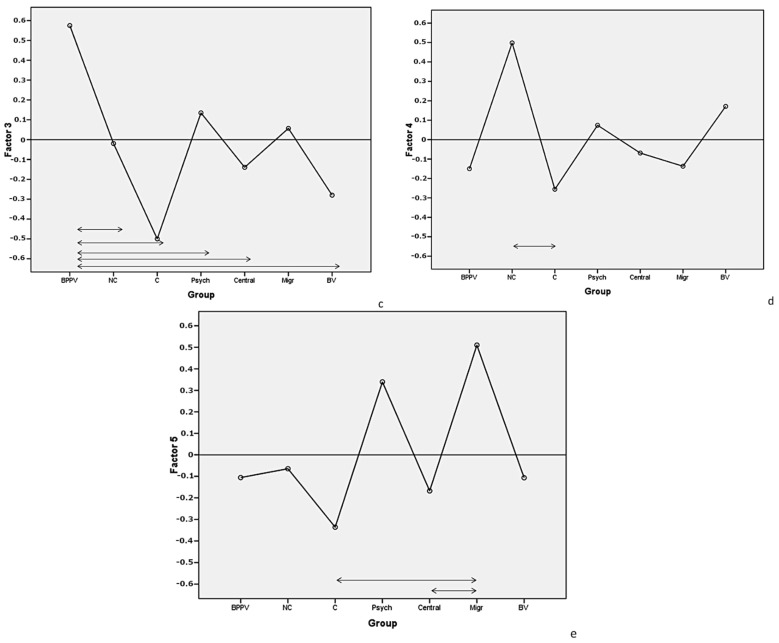
The relationships between clinical groups and DHI factors. (**a**–**e**) present the scoring for clinical groups for factors F1, F2, F3, F4, and F5, respectively. Arrows show the statistically significant differences between scores in clinical groups. BPPV—benign paroxysmal positional vertigo. NC—vestibular noncompensated group. C—vestibular compensated group. Migr—migraine group. BV—bilateral vestibular impairment. Psych—psychogenic dizziness.

Factor 3 revealed scoring significantly higher in the BPPV group. Factor 4 differentiated the exclusively compensated and noncompensated vestibular patients, while Factor 5 was the highest scoring in the migraine group (Figure 3a–e).

### 3.3. The Clinical Utility of the Factors

Receiver operating characteristics (ROC) curve analysis was performed to evaluate the clinical meaning of the relationships revealed by the intergroup factor analysis. The ROC analysis confirmed the differences between the noncompensated (NC) and compensated (C) vestibular groups for the DHI total score: Area under curve (AUC) 0.74, *p* = 0.0000; positive predictive value (PPV) 76%, negative predictive value (NPV) 68%, cut point 52.

PCA revealed statistically significant differences between NC and C groups in F2 and F4. The ROC analysis confirmed the relationships: F2—PPV 79%, NPV 62%, cut point 14, AUC 0.75 *p* = 0.0000; F4: PPV 76% and NPV 60%, AUC 0.70, *p* = 0.0003, cut point 8. The values of AUC for F1, F3, and F5 were markedly lower (below 0.65) even if the probability of models was statistically significant.

The F3 group of items presented the highest scoring in the BPPV subgroup. At the value of 14 points, the sensitivity was 75% but the specificity was 54%, PPV was 23% and NPV was 92%, and AUC was 0.66 (*p* = 0.0000).

The F5 was highly corelated with depression and anxiety. The ROC analysis revealed a high sensitivity of F5 (85%) in the psychogenic group in relation to the remaining population tested; however, the specificity was low (42%, AUC 0.67, *p* = 0.0000). NPV was 88% and PPV was 36% in that group.

## 4. Discussion

The main aim of the present study was to identify the individual subscales of the DHI, which would correlate with clinical tests in a cohort of patients with vestibular and nonvestibular vertigo or dizziness. We were interested in finding whether the subscales of DHI could be used as an indicator of a clinical condition (e.g., compensation, positional vertigo).

The DHI results obtained from a cohort of patients with vestibular and nonvestibular diseases were calculated using factor analysis. The first step was to identify individual subscales of DHI; then, we conducted a comparative analysis of the results between the groups of patients with vertigo and nonvestibular dizziness or vertigo.

### 4.1. Subscales Reliability

The exploratory factor analysis revealed five factors with eigenvalues >1, which explained 58% of the variance, which is similar to Kurre et al. (54.5%) [7]. Two items that overlapped F2 and F3 factors may lower this percentage. Before rotation, only two factors were obvious; the former included almost all the items and the latter only the P13 and F5. Matching results were reported by Asmundson et al. [23]. After oblique rotation, five factors fulfilling the assumptions were extracted.

Factor 1 contains five items connected to restricted participation, such as restriction of travel, social activities, walking, or staying home alone. Of all the factors, F1 reveals the highest Cronbach’s alpha. Three of these five items were extracted by Kurre et al. [7] as Factor 4, which has been rejected by the authors. Tamber et al. [8] extracted four of our five items as Factor 3. Perez et al. [10] in Factor 1 named ‘vestibular handicap’ found all items from our F1 (restriction of participation), adding F4 (handicap/anxiety) and F5 (depression). To some degree, the factors 1, 4, and 5 are comparable in their meaning but, in contrast to Perez et al. [10], we found differences between them during clinical group analysis.

Factor 2 contained items that are connected to activities aggravating the vestibular symptoms (walking in darkness, ambitious activities, avoiding heights) and visual overdependence (walking through supermarkets, walking down sidewalks). Two items in this factor are ambiguous. The first ambiguous item is (F)12, which some subjects interpreted as a fear of heights in the mountains. The second item—(P)17 (walking on the sidewalk)—is ambiguous. Most of the healthy subjects were interpreting it as walking on uneven pavement, similarly to Sousa et al. [24], while the vestibular patients underlined the problems with movement when generally walking outside in the traffic. Our Factor 2 is almost completely in agreement with Factor 4.3 (contextual factors or effort provoking dizziness and unsteadiness) reported by Kurre et al. [7] and Factor 3 (visuo-vestibular disability) reported by Perez et al. [10].

Factor 3 encloses items that are characteristic for positional vertigo. These symptoms were also separated by Kurre et al. [7] in Factor 4.2 and Asmundson et al. [23] but not by Perez et al. [10] or Tamber et al. [8]. Kurre et al. [7] added the P1 item to this factor, which in our study, was between the vestibular (F2) and positional (F3) symptoms. Most of the previous analyses were performed for the vestibular subjects, whereas the central and psychological disorders were excluded. In one study by Yip and Strupp [15] in which the peripheral, central, positional, and psychogenic groups of patients were analyzed, the results were closely related to ours.

Factors 4 and 5 include the items mainly connected with handicap (F4) and anxiety/depression (F5). F5 was highly correlated to Duke anxiety/depression questionnaire scores. Kurre et al. [7] combined these items into one (effect of dizziness and unsteadiness on emotion). However, in our study, the Cronbach’s alphas calculated separately for two factors yielded acceptable results, while after combining them into one, the resultant Cronbach’s alpha was <0.7, thus diminishing the internal consistency. The factors F3 and F4 consist of items originally assigned to the subscale E (emotional), but the relationships between these factors and the Duke anxiety/depression questionnaire were markedly lower.

### 4.2. Analysis in Clinical Subgroups

The DHI distribution across the clinical groups demonstrated the lowest mean values of scores in the vestibular compensated group. The DHI total score differentiated between the compensated and noncompensated with a 76% positive predictive value. This means that 76% of the patients recognized as uncompensated in the rotational chair test (VNG) had the DHI scoring above 52 points. In factor analysis, the compensated group revealed the lowest result in Factor 2, devoted to the vestibular symptoms. This factor showed quite a high positive predictive value of 79% to differentiate between the noncompensated and compensated groups.

The Dizziness Handicap Inventory, Visual Analogue Scale, Falls Efficacy Scale, and the Vertigo Symptom Scale are the most commonly used self-reported patients outcome measures in vestibular rehabilitation [25].

However, it is assumed [12] that the vestibular symptoms described in DHI are nonspecific for any disease. The compensation processes are monitored by objective measurements of vestibulo-ocular reflex parameters or balance system assessment [26].

The previous studies reported correlations between the DHI scores and the results of dynamic posturography [6,20], and functional tests [27], but there are little data on the relationships between the handicap or symptoms intensity and objective tests results [6]. Some authors stated that the symptoms intensity and disability level hardly correlate with the objective tests results that are used in the assessment of the compensation [15,28]. Yip and Strupp [15] have not confirmed any relationships between the DHI and quantitative vestibular tests such as video head impulse test, caloric test (VNG), or cervical and ocular vestibular-evoked myogenic potentials, but they did not consider rotational chair tests in their analyses. Thus, our findings give the opportunity to propose such a simple tool as a questionnaire for monitoring the compensation process.

In our study, we assess the usefulness of the DHI in BPPV evaluation. F3 reaches the highest mean scoring in the BPPV subgroup and quite a high sensitivity of 75%. However, a high value of false positive results suggests that these symptoms are reported by patients diagnosed with the vestibular, psychogenic migraine status (Figure 3c). The high sensitivity of the positional subscale (81%) was also calculated by Whitney et al. [12]. In the Whitney et al. study, similarly to ours, the specificity of the positional subscale was low. The BPPV underdiagnosis seems to be a common problem [29]. Any attempt to improve diagnostics is of great importance when referring patients to the appropriate units. Our data may be used to confirm that there is a group of DHI items that are quite specific to the BPPV (but not only to this clinical condition) and to suspect that low scoring of these items may exclude positional vertigo.

The DHI total score and all subscales were correlated with the anxiety-depression scale. The highest values of the DHI score were observed in the vestibular noncompensated group but also in the psychogenic group without vestibular impairment (Figure 2). The highest correlation coefficient was found between the Duke anxiety-depression scale and DHI vestibular (F2 0.56) and anxiety-depression (F5 0.51) factors. The group of items included with F5 revealed a sensitivity of 85% differentiating the psychogenic group from others. A high disability level was observed in patients with functional neurological (psychogenic) conditions without any vestibular disorder. Our results are in line with those obtained by Yip and Strupp [15], who also found a significantly higher DHI in patients with primary functional dizziness than in patients with stable vestibular dysfunction. Moreover, some authors stated that the psychogenic conditions may exacerbate the vestibular symptoms [18,19]. The high correlation of the vestibular subscale to the Duke anxiety-depression scale confirmed the observations reported by Piker et al. [19].

Central as well as the bilateral vestibular subgroups did not reveal any correlation to the DHI subscales. The migraine group showed the highest scoring for anxiety-depression items (Figure 3e). The relationship between migraine and anxiety-depression has been confirmed by other authors [30].

The main strength of the study is access to a large database. This allowed the choice of relatively numerous subgroups of subjects with a single clinical condition. Usually, the main problem in clinical studies concerns the co-occurrence of diseases in the same person, e.g., anxiety in dizzy patients, BPPV and vestibular loss, vestibular and central signs in VNG recordings.

## 5. Conclusions

1. The DHI questionnaire total scoring and its vestibular subscale distinguish between the patients with compensated and uncompensated vestibular dysfunction with positive predictive values of 76% and 79%, respectively, which may be clinically useful for monitoring and modifying rehabilitation therapy.
2. The DHI items composing the F3 (positional) subscale revealed the highest scoring in the BPPV group. However, these items are not exclusively devoted to positional vertigo. The low scoring of positional items may suggest any other reason besides BPPV that causes the patient’s symptoms.
3. The DHI total scoring and subscales were correlated with anxiety and depression. The anxiety and depression subscale (F5) revealed the highest correlation and a high sensitivity in the psychogenic group. This subscale may be helpful in considering the functional neurological condition as the main problem of a dizzy patient.

## Figures and Tables

**Table 1:** DHI principal component analysis with oblique rotation factors. Labels and loadings of the corresponding item, the original subscales labels are in the brackets.

Items	Title	Factor 1	Factor 2	Factor 3	Factor 4	Factor 5
		restricted participation	vestibular	Positional	handicap	Anxiety, depression
(F)3	Restriction of travel	0.783				
(F)6	Restriction of social activities	0.855				
(F)16	Walking by yourself	0.653				
(E)20	Afraid of staying home alone	0.618				
(E)9	Afraid of leaving home alone	0.589				
(E)22	Family relationships	0.486				
	**Cronbach’s alpha**	**0.828**				
(P)4	Walking through supermarkets		0.790			
(P)8	Ambitious activities		0.764			
(P)17	Walking down sidewalks		0.593			
(F)19	Walking in darkness		0.535			
(F)12	Avoiding heights		0.430			
	**Cronbach’s alpha**		**0.777**			
(P)1	Looking up		0.389	0.384		
(F)7	Difficulties reading		0.241	0.242		
(F)5	Getting in/out of bed			0.921		
(P)13	Turning over in bed			0.960		
(P)11	Quick head movements			0.506		
(F)14	Strenuous home work			0.353		
(P)25	Bending over			0.391		
	**Cronbach’s alpha**			**0.734**		
(E)15	Afraid of appearing drunk				0.895	
(E)10	Feeling embarrassed				0.685	
(E)21	handicapped				0.631	
	**Cronbach’s alpha**				**0.728**	
(E)2	Frustrated					0.709
(E)23	Feeling depressed					0.607
(E)18	Difficulty concentrating					0.543
(F)24	Job/house responsibilities					0.505
	Cronbach’s alpha					**0.715**

## Data Availability

The data presented in this study are openly available in repository https://ppm.imp.lodz.pl/info/researchdata/IMPe342ef80d65742fc8926f4c7a427db5b/ (accessed on 24 February 2021).
